# Resolving the metabolon: is the proof in the metabolite?

**DOI:** 10.15252/embr.202050774

**Published:** 2020-08-04

**Authors:** Youjun Zhang, Alisdair R Fernie

**Affiliations:** ^1^ Center of Plant Systems Biology and Biotechnology Plovdiv Bulgaria; ^2^ Max‐Planck‐Institute of Molecular Plant Physiology Potsdam‐Golm Germany

**Keywords:** Metabolism

## Abstract

Metabolons are supra‐molecular complexes of metabolic enzymes and cellular structural elements. Even though the term was coined 35 years ago, the existence of metabolons was only recently demonstrated by a combination of metabolomics and state‐of‐the‐art mass spectrometry.

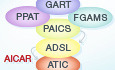

It is now 35 years since Paul Srere coined the term metabolon to define “supra‐molecular complexes of sequential metabolic enzymes and cellular structural elements” (Srere, 1985). However, despite massive technological advances in biochemistry, molecular biology, and genetics, the detection of metabolons within the cell remains as challenging as it was 35 years ago. What is most commonly demonstrated is merely co‐clustering or at best the physical interaction of enzymes, which many authors—erroneously in our opinion—seem to regard as sufficient to label as a metabolon. Such interactions would be more aptly described as enzyme–enzyme assemblies since the experiments do not demonstrate whether the enzymes mediate substrate channeling.

As we will discuss below, demonstrating this is difficult. Historically, researchers have been using methods based on (i) transient time analysis; (ii) comparison of enzyme activity in the presence of a competing enzyme; (iii) comparison of the activity of an enzyme pair in the presence of an inhibitor of the second enzyme of the pair; (iv) enzyme buffering; and (v) isotope dilution experiments (Fernie *et al*, 2018). A recent study by Vidhi Pareek *et al* used metabolomics and state‐of‐the‐art gas cluster beam secondary ion mass spectrometry (GCIB‐SIMS) to directly visualize *de novo* purine biosynthesis in the enzyme–enzyme assembly known as the purinosome (Pareek *et al*, 2020) as an alternative method to study metabolite channeling. Since we regard proof of channeling essential for calling an enzyme–enzyme assembly a metabolon, we will describe both methods in more detail.

Given the complexity of these methods, we advocate their use only after using cell biology, proteomics, or electron microscopy to provide structural evidence of assembly. The amount of protein–protein interactions within a cell is staggering: Screens of human cells suggest up to 130,000 binary interactions at any one time (Fernie *et al*, 2018). Recent years have seen massive technological developments, such as FLIM‐FRET and BRET alongside advanced proteomics methodologies, which make the identification of enzyme–enzyme assemblies relatively facile (Laursen *et al*, 2016; Zhang *et al*, 2017; McWhite *et al*, 2020). However, the quantitative determination of the relative amounts of bound versus free enzymes is rarely reported rendering their biological importance open to interpretation.

Furthermore, whether enzyme–enzyme interactions act to compartmentalize and even channel metabolites is not possible to ascertain from such methods. Structural information from crystallography and electron microcopy (Yu *et al*, 2020) can provide evidence—or lack thereof—of channeling. However, such data are, at least to date, also largely qualitative, thereby limiting our ability to interpret the functional consequence of the reported structures. Thus, while highly informative and even perhaps illustrative of underlying mechanisms, these techniques alone do not provide sufficient evidence of a functional metabolon. We argue that additional metabolite‐level information is required.

One approach to do this is almost as old as the concept itself: analyzing isotopic enrichment of a pathway product as a function of isotope‐labeled substrate and unlabeled metabolic intermediates. Such experiments were used by the Sumegi and Srere groups to study primary metabolism (Sumegi *et al*, 1991) and in early studies of the role of metabolons in plant metabolism (Møller & Conn, 1980). Recently, such experiments demonstrated the evolutionarily conserved role of the metabolon in glycolysis and the TCA cycle (Zhang *et al*, 2017) and that parts of the purinosome complex do indeed operate as a metabolon (Pareek *et al*, 2020). The latter study additionally used ultra‐high‐resolution mass‐spectral‐imaging technique to measure the concentration of metabolites within the direct locality of the enzymes to complement, albeit less rigorously, the evidence of channeling provided in their isotope‐based studies.

Purine nucleotides are key constituents of DNA and RNA, energy carriers, and cofactors. Intriguingly, if the cell has a high demand for purines, the enzymes of the *de novo* purine biosynthesis cluster together into multi‐enzyme complexes which have become known as purinosomes. As reviewed in (Sweetlove & Fernie, 2018), confocal microscopy of HeLa cells revealed co‐location of all six enzymes of the pathway in clusters of 0.2–0.9 μm in diameter under conditions of high purine biosynthesis. Moreover, proteomics demonstrated that the core complex assembles in a stepwise fashion to include the first three enzymes in the pathway; further congregation of the purinosome requires the Hsp70/Hsp90 chaperone machinery, casein kinase, microtubules, and G‐protein‐coupled receptors. Given its size, it was previously suggested that the purinosome contains hundreds of the three core‐complex enzymes, brought together by interactions with the remaining pathway enzymes (Sweetlove & Fernie, 2018).

While this evidence has been used to claim the purinosome is a metabolon, it is still circumstantial. In their current study, the Benkovic group provide direct proof by demonstrating that the six‐enzyme, two‐metabolite pathway within the purinosome is functional and that it promotes substrate channeling (Fig 1). While the best proof of the latter comes from their isotope tracing experiments, the quantitative analysis performed by the ultra‐high‐resolution mass‐spectral‐imaging technique further provides strong support.

**Figure 1:**
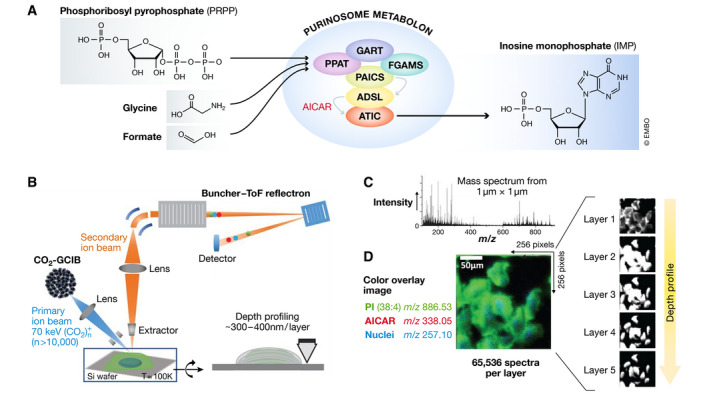
Identification of purinosome metabolon by *in situ* GCIB‐SIMS (A) The purinosome metabolon contains six enzymes that channel metabolites to produce purines. Using the phosphoribosyl pyrophosphate, as well as formate and glycine, it synthesizes inosine monophosphate. (B) Schematic of GCIB‐SIMS imaging of HeLa cells. Imaging uses a finely focused 70‐keV (CO_2_)_*n*_^+^ (*n* > 10,000) cluster beam to interrogate frozen‐hydrated HeLa cells three‐dimensionally at 1‐μm spatial resolution. Coupled with a buncher‐ToF and direct‐current beam setup, maximum spatial resolution and mass resolution can be retained. A pixel‐by‐pixel analysis was performed across a lateral field of view of 256 μm × 256 μm. (C) Mass spectra in the *m/z* range 0 to 900 were recorded for each pixel. (D) A composite two‐dimensional colored image was generated combining the signal across all the layers PI (38:4; green) at *m/z* 886.53, phosphate‐sugar backbone at *m/z* 257.10 (blue) from nucleotides, and ^15^N‐enriched DNPB intermediate AICAR (red). Combination of mass spectral analysis and the spatial distribution of specific cellular signals demonstrates the reliability of the method for *in situ* biochemical studies (Srere, 1985). With permission by AAAS. PPAT, phosphoribosylpyrophosphate amidotransferase; ADSL, adenylosuccinate lyase; ATIC, 5‐aminoimidazole‐4‐carboxamide ribonucleotide formyltransferase/5′‐inosinemonophosphate cyclohydrolase; FGAMS, phosphoribosylformylglycinamidine synthase; GART, phosphoribosylglycinamide synthetase; PAICS, phosphoribosyl aminoimidazole succinocarboxamide synthetase.

Beyond the purinosome, adopting this approach to study enzyme–enzyme assemblies such as the TCA cycle, glycolysis, and cyanogenic glucoside biosynthesis for which strong evidence of metabolite channeling already exists and, perhaps more importantly, pathways for which this is currently lacking will greatly enhance our understanding of how common channeling is within metabolism. Such analyses also open up manifold possibilities for studying the mechanisms underlying metabolon association and dissociation, and the regulation of metabolons in response to, for example, stress, disease, and developmental transitions. Finally, the question whether the formation of metabolons is quantitatively dependent on chemotactic gradients—an observation from cell‐free microfluidic systems (Laursen *et al*, 2016)—holds true *in vivo* could also be addressed by this approach.

In summary, Pareek *et al* (Srere, 1985) demonstrate a further route to evaluating the functional roles of transient enzyme–enzyme assemblies which do and do not mediate metabolite channeling. It is our strong contention that both are biologically relevant, but only the latter are metabolons as described by Srere all those years ago. The direct imaging of metabolite concentrations will certainly greatly enhance our understanding of these enigmatic structures.
